# Reorientation methodology for reproducible head posture in serial cone beam computed tomography images

**DOI:** 10.1038/s41598-023-30430-4

**Published:** 2023-02-24

**Authors:** Utkarsh Mangal, Sung Min Lee, Seeyoon Lee, Jung-Yul Cha, Kee-Joon Lee, Hyung-Seog Yu, Hong Jung, Sung-Hwan Choi

**Affiliations:** 1 Department of Orthodontics, Institute of Craniofacial Deformity, Yonsei University College of Dentistry, Seoul, 03722 Korea; 2 HDXWILL, Seoul, 03162 Korea

**Keywords:** Cone-beam computed tomography, Digital radiography in dentistry

## Abstract

Low dose and accessibility have increased the application of cone beam computed tomography (CBCT). Often serial images are captured for patients to diagnose and plan treatment in the craniofacial region. However, CBCT images are highly variable and lack harmonious reproduction, especially in the head’s orientation. Though user-defined orientation methods have been suggested, the reproducibility remains controversial. Here, we propose a landmark-free reorientation methodology based on principal component analysis (PCA) for harmonious orientation of serially captured CBCTs. We analyzed three serial CBCT scans collected for 29 individuals who underwent orthognathic surgery. We first defined a region of interest with the proposed protocol by combining 2D rendering and 3D convex hull method, and identified an intermediary arrangement point. PCA identified the y-axis (anterioposterior) followed by the secondary x-axis (transverse). Finally, by defining the perpendicular z-axis, a new global orientation was assigned. The goodness of alignment (Hausdorff distance) showed a marked improvement (> 50%). Furthermore, we clustered cases based on clinical asymmetry and validated that the protocol was unaffected by the severity of the skeletal deformity. Therefore, it could be suggested that integrating the proposed algorithm as the preliminary step in CBCT evaluation will address a fundamental step towards harmonizing the craniofacial imaging records.

## Introduction

Digital imaging has expanded the diagnosis and treatment planning scope in dentistry. Cone beam computed tomography (CBCT) has become the imaging technique of choice that provides high-resolution 3-dimensional (3D) volumetric data for facial morphology analysis^1,2^.

The most critical aspect of 3D cephalometric assessment is the positioning of the skull in space to allow an appropriate definition of the anatomical frame for the craniofacial region^3^. A common practice in clinically acquiring radiographic images is to subjectively orient the patient head along an imaginary plan with reference from nose to ear (ala-tragal line)^4^. In 3D CBCT imaging, longer-duration scans (20–40 s) are performed to capture the complete craniofacial region^5^. As the image quality is critically dependent on the stability of the head, external guides are used, which are arbitrary and do not conform to the patient’s orientation. Moreover, patient head orientation can also vary from lying down to sitting to standing, depending on the CBCT scanner used^6^. Although an extracranial reference can be used during the scan, the transfer of the reference element to the 3D-rendered visualization of the head volume cannot be reproducible^7,8^. Because of the above reasons, a user-defined approach is used to reorient the radiographic images, which involves labeling anatomic and derived landmarks. Regardless of the type of imaging modality, 2D or 3D, skull positioning relies on the horizontal and vertical reference planes derived from the manually labeled coordinates.

The Frankfurt horizontal (FH) plane is the most practiced horizontal reference plane formed by identifying two bilateral landmarks (porion and orbitale)^9^. However, the FH plane is vexed by the highly error-prone nature of its non-coplanar representative landmarks^10^. Additionally, the construction of the FH plane has competing descriptions in literature, reducing the reproducibility within the subjects^11^. The drawbacks of the FH plane have been addressed in literature with suggestions of alternate horizontal and vertical planes^12^. Basion, sella, nasion, and pogonion have been proposed in various combinations to define the true vertical plane, which can orient the skull in 3D space for effective diagnosis^13^. However, most alternative reference planes involve a significant manual landmark-based assignment which is disadvantaged by large inter–and intra–examiner variability. Together with the inherent variations in CBCT images, the manual orientation method fails to harmonize the serial CBCT images for effective and reproducible clinical use^14^.

Findings from previous studies simulating variation in head orientation during CBCT scans have suggested only mild variation in the reliability of measurement. However, the study designs are either arbitrary with fiducial markers or based on dry skull models^5,15,16^. Although lately different methods for head orientation in 3D space have been proposed, they have either been focused on 3D facial scans or based on high-resolution medical CT images^4,17–20^.

**Figure 1:**
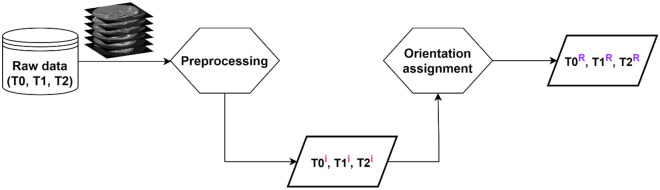
Scheme of the proposed approach for processing serial 3D cone beam computed tomography (CBCT) volumes for a reproducible landmark-free reorientation. T0, T1, and T2 represent serially captured CBCT volumes. Superscripts *i* and *R* indicate input records for orientation assignment and the output reoriented images, respectively.

CBCT images are a valuable tool for 3D characterization, and serial images form a part of treatment and follow-up plans in orthodontic and surgical cases. We present a landmark-free reproducible head orientation protocol in serial CBCT images. We describe a two-step approach using principal component analysis (PCA) to orient the skull in serially captured CBCTs from patients surgically treated for facial asymmetry (Fig. 1). Lastly, we present the improvement in the goodness of alignment by application of the proposed reorientation method in three sequential CBCT volumes by Hausdorff distance (HD) metric.

## Methodology

### Study design

The retrospective study protocol conformed to the Declaration of Helsinki and was approved by the institutional review board of Yonsei University Dental Hospital (2-2022-0073) and passed the exemption review for informed consent on the use of patients’ radiography and medical records. The study sample was acquired from the Department of Orthodontics archives of the adult patients who underwent orthognathic surgical therapy between 2010 and 2018 at Yonsei University Dental Hospital, Seoul, Republic of Korea. The inclusion criteria for the study were: (1) age > 18 years; (2) history of clinical diagnosis of skeletal asymmetry defined by the presence of ≥ 4 mm deviation of the menton (anteroinferior point on the chin) from the facial midsagittal plane^21^; and (3) availability of serial CBCT data sets of a large field of view (FOV) and a voxel size ≤ 0.4 mm. The exclusion criteria were (1) history of maxillofacial trauma, (2) CBCT images of insufficient quality due to motion blurring artifacts, and (3) difference of FOV between serial CBCT images.

A total of 57 records with multiple serial CBCT images were identified, from which 29 records that met the inclusion criteria were selected (19 males and 10 females; mean ± SD age, 22.10 ± 3.22y; range, 18–32y). Each of the selected records had a minimum of three serial CBCT volumes. The CBCT volumes were then categorized as T0, T1, and T2. Here, T0 was the first CBCT volume recorded for an individual, with T1 and T2 being the second and third serially collected volumes.

### Image preprocessing

All CBCT scans were acquired under the same parametric settings at standard operational settings of the hospital (80 kVp; 10 mA) with Alphard3030 (Alphard Roentgen Ind., Ltd., Kyoto, Japan).

The voxel resolution of 0.39 mm for a 200 mm × 200 mm field of view was acquired with a maximum scan time of 17 s. The obtained scan data was accessed in digital imaging and communication in medicine (DICOM) format and anonymized before further computational processing. The algorithm proposed was implemented and tested by Python 3.7.13 on a CPU (Intel(R) Core (TM) i7-10750H, 2.60 GHz) and GPU (NVIDIA GeForce RTX 3060. 8 GB) system.

**Figure 2:**
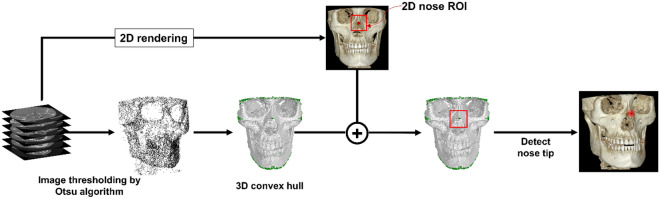
Schematic representation of the initial preprocessing step. This step determines the outermost projection in the naso-orbital region within the three-dimensional segmented images.

Figure 2 provides a graphical representation of the preprocessing step from the multi-stack raw DICOM data. The preprocessing step defines a region of interest (ROI) that forms the main operating region for subsequent orientation assignments. The DICOM data is 2D rendered, and ROI is defined in the naso-orbital region. With the help of Otsu’s thresholding algorithm, the noise and soft tissue were eliminated for extraction of the dentoskeletal region^22^. A 3D convex hull was then processed over the segmented skull images. Because the skull is concave within a limited range except near the nose tip, there are no elements of the convex hull in the vicinity except near the apex of the nasal region, like the green dot in Fig. 2. Therefore, among all convex hull elements whose x, z coordinates are within the 2D nose ROI, only the points near the nasal projection can be selected. The nose tip can be uniquely defined by obtaining the average of these points. Hence, using together the information from 2D ROI and the result from the convex hull, automated identification of the highest projection point within the naso-orbital region of the skull was made. This point is referred to as ‘nose tip’ from here on.

### Intermediary arrangement

The described preprocessing approach was applied individually to each serially obtained CBCT image. An intermediary’ arrangement’ was performed to harmonize the orientation between the serial images. In this step, the CBCT volumes were arranged with the nose tip as the reference before the new global axis was defined. This step enables an improved agreement between the new global axes of the multiple serial volumes, as detailed in the next section.

### Orientation assignment

**Figure 3:**
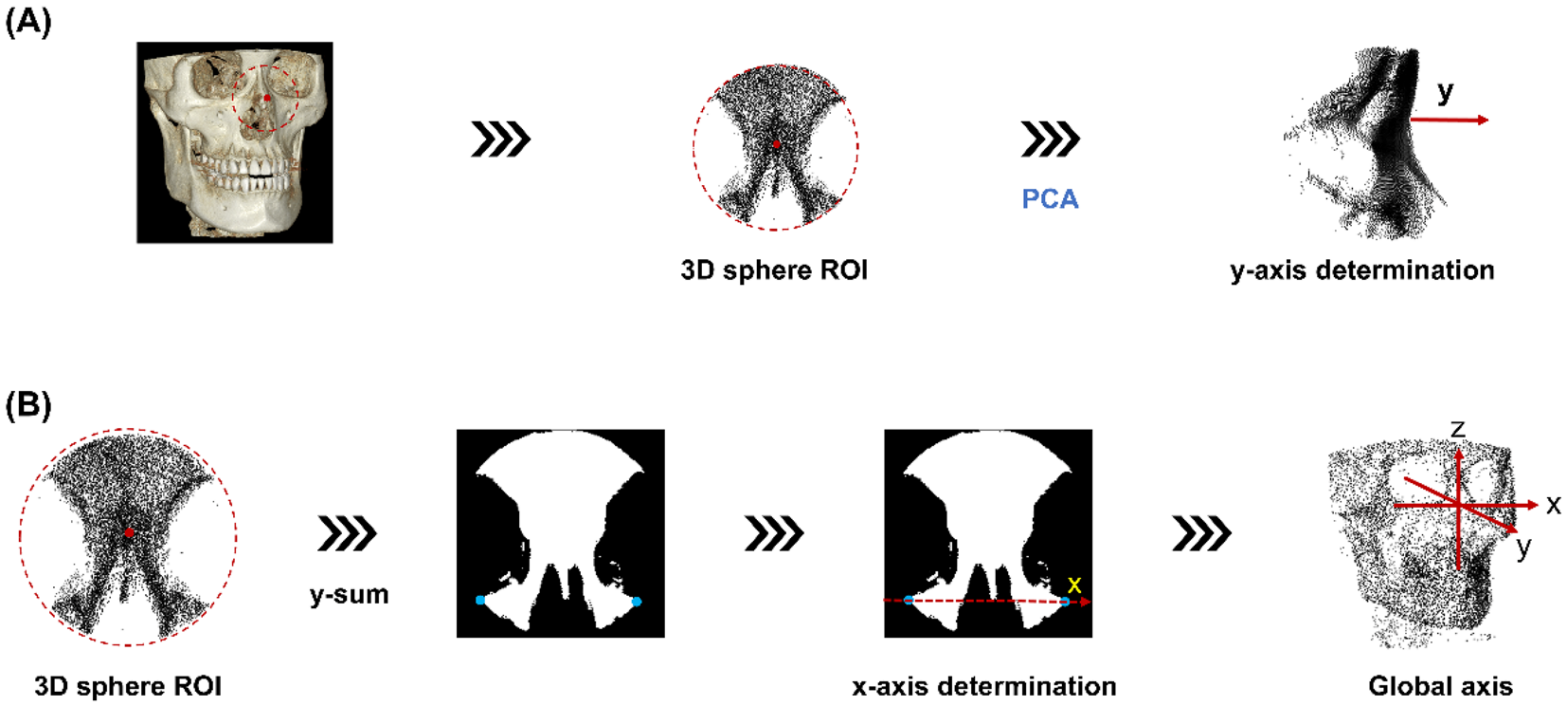
Orientation assignment. Determination of the (**A**) y-axis followed by (**B**) x-axis determination from the 3D spherical region of interest (ROI). The global axis orientation assignment is completed with a z-axis definition.

In the segmented skull image, 3D sphere ROI is defined as a sphere with a radius of 35 mm centered on the nose tip obtained in the previous step. The point distribution in the 3D spherical ROI, centered on the tip of the nose, appears larger along the face of the skull and smaller in the direction corresponding to the depth of the skull. That is, the axis with the smallest PCA component of the 3D skull ROI is in the depth of the skull, which represents the anteroposterior head orientation. Therefore, we defined the new y-axis as the axis with the smallest component of PCA (Fig. 3A).

A 2D image is obtained by summation of the 3D spherical ROI of the skull in the newly determined y-axis direction. Binarization is performed by setting the region with a value greater than 0 to 1. In the 2D image, with only the image of the bottom half from the center, the two points at the far ends of the x-axis are automatically selected. The direction of the straight line passing through the two points (blue) is determined as the new x-axis, as shown in Fig. 3B. A third perpendicular axis, the z-axis, was defined with the newly obtained x and y axes allowing the completion of the 3D global axis. With the help of the redefined 3D global axis, CBCT reorientation was completed.

### Evaluation measures

#### Improvement in goodness of alignment

**Figure 4:**
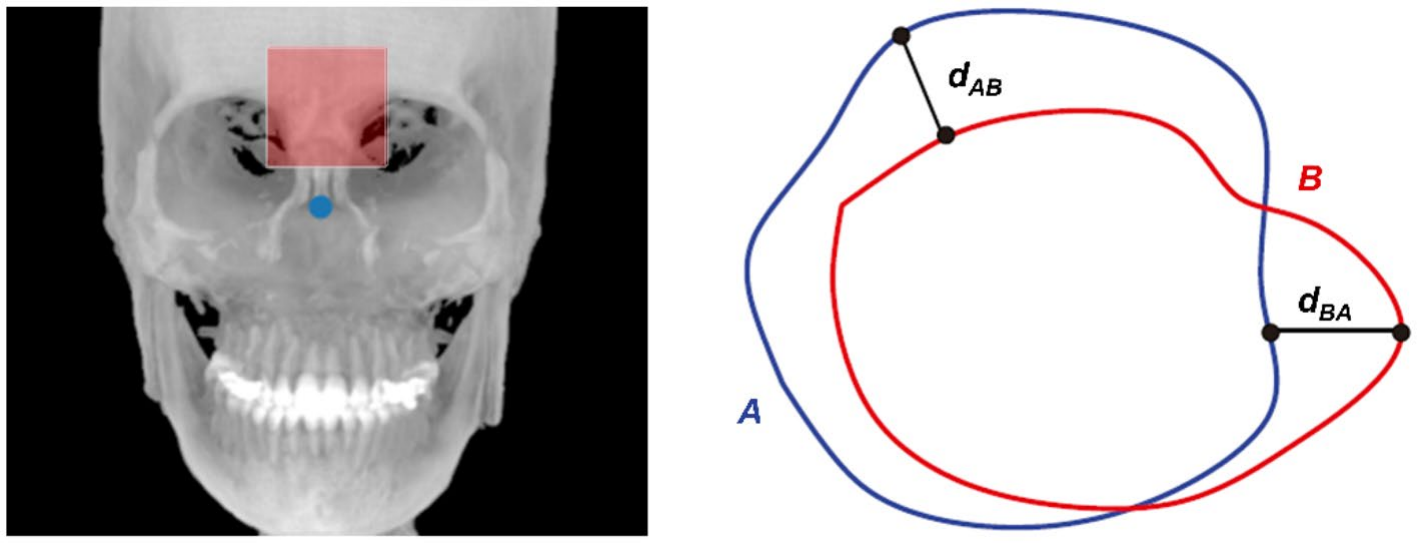
A schematic showing the Hausdorff distance between points sets *A* and *B* from the forehead surface (red shaded box).

We performed a quantitative and qualitative evaluation of the changes in the serial CBCT images. The quantitative changes were evaluated by comparing the angular changes with the redefined 3D global axis. The assessment of the general alignment between serial images with the HD was performed and computed for serial image pairs: T0T1, T0T2, and T1T2 using Eq. (1)

1 $$HD \left( {A,B} \right) = max\left[ {{\text{min}}_{A} \left\{ {d\left( {A,B} \right)} \right\}, {\text{min}}_{B} \left\{ {d\left( {B,A} \right)} \right\}} \right]$$

where *d* stands for the 3D Euclidean norm, and A and B represent the two sets derived from the forehead surface (Fig. 4), such that a lower HD value indicates the goodness of alignment^23,24^.

Changes in the HD value after processing via the proposed protocol were compared, herein referred to as ‘matching improvement.’ Additionally, the impact of the intermediary step was evaluated by comparing the HD changes with and without the arrangement.

#### Comparing goodness of alignment based on severity of asymmetry

With K-medoids clustering, the original data were clustered into three groups based on the clinical magnitude of asymmetry: mild (4–4.5 mm), moderate (4.5–9.5 mm), and marked (> 9.5 mm). The groups were then compared for HD matching improvement after the PCA-based reorientation to evaluate the pairwise goodness of alignment as described above. Lastly, qualitative visualization of the images was performed to depict the summed alignment of the serial CBCT volumes.

## Results

### Rotational changes with new axis definition

**Figure 5:**
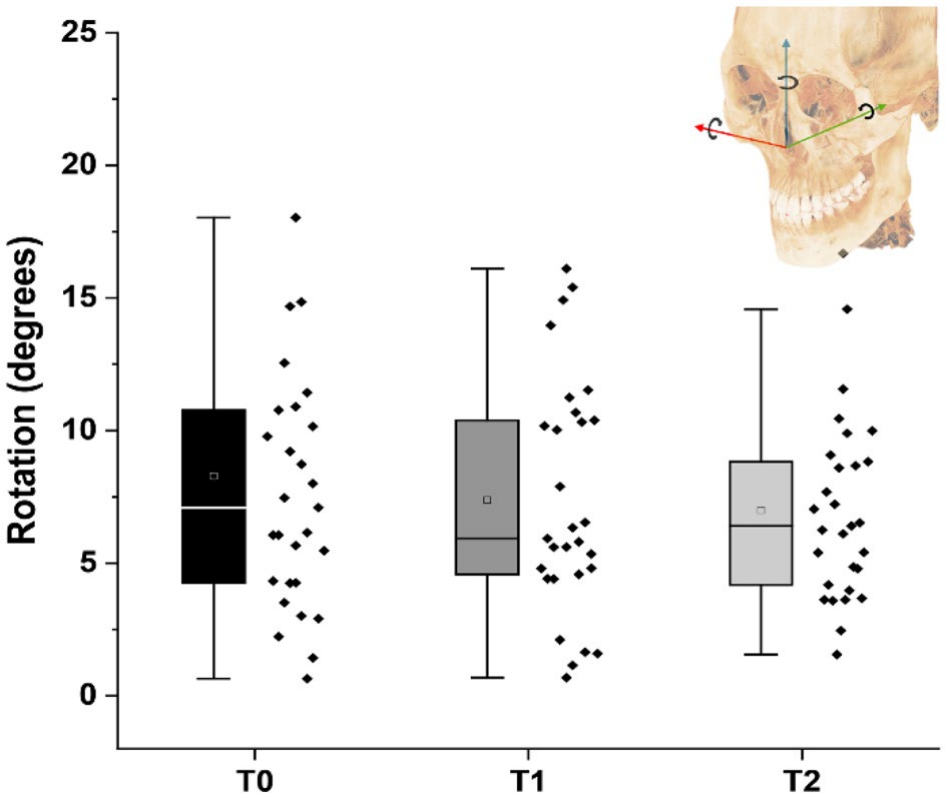
Changes in the orientation of the cone beam computed tomography head images observed at different time points.

Figure 5 presents the angular changes following the orientation assignment at each of the three-time points. The rotational changes (mean ± standard error) were observed to be similar between T0 (8.28 ± 1.12 degrees), T1 (7.37 ± 0.82 degrees), and T2 (6.98 ± 0.65 degrees). The similar effect with the new global axis, regardless of the stage of CBCT volume capture, indicated that the computational approach is independent of a preexisting reference. The axes of rotation are detailed in Table S1.

### Effect of intermediary arrangement step

**Figure 6:**
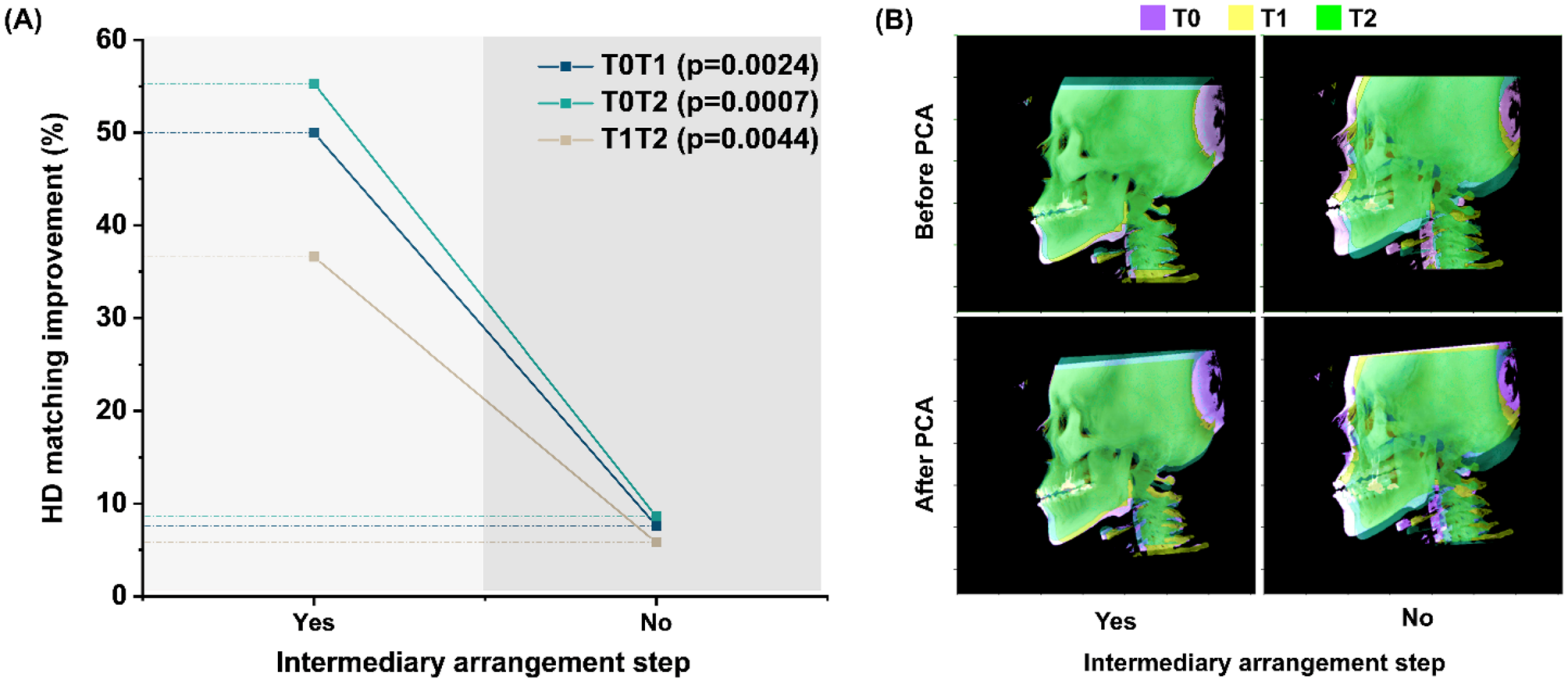
Comparison of the goodness of alignment of serial CBCT volumes with the intermediary arrangement step performed before the global axis is defined by measuring the (**A**) Hausdorff distance (HD) and (**B**) image overlay. PCA, principal component analysis.

The intermediary arrangement step, which arranged the serial images based on nose tip point reference, significantly improved the HD metric. Figure 6A presents a statistically significant difference observed with Wilcoxon pairwise analysis between the different sets of CBCT volumes. Visualization of the three serial images from a mild clinical asymmetry group also shows a marked difference in the orientation of the volume. It indicates that PCA computation without an ROI marker arrangement can be erroneous and lack reproducibility in serially captured CBCT volumes (Fig. 6B).

### Quantitative improvement in alignment between serial CBCT volumes

**Figure 7:**
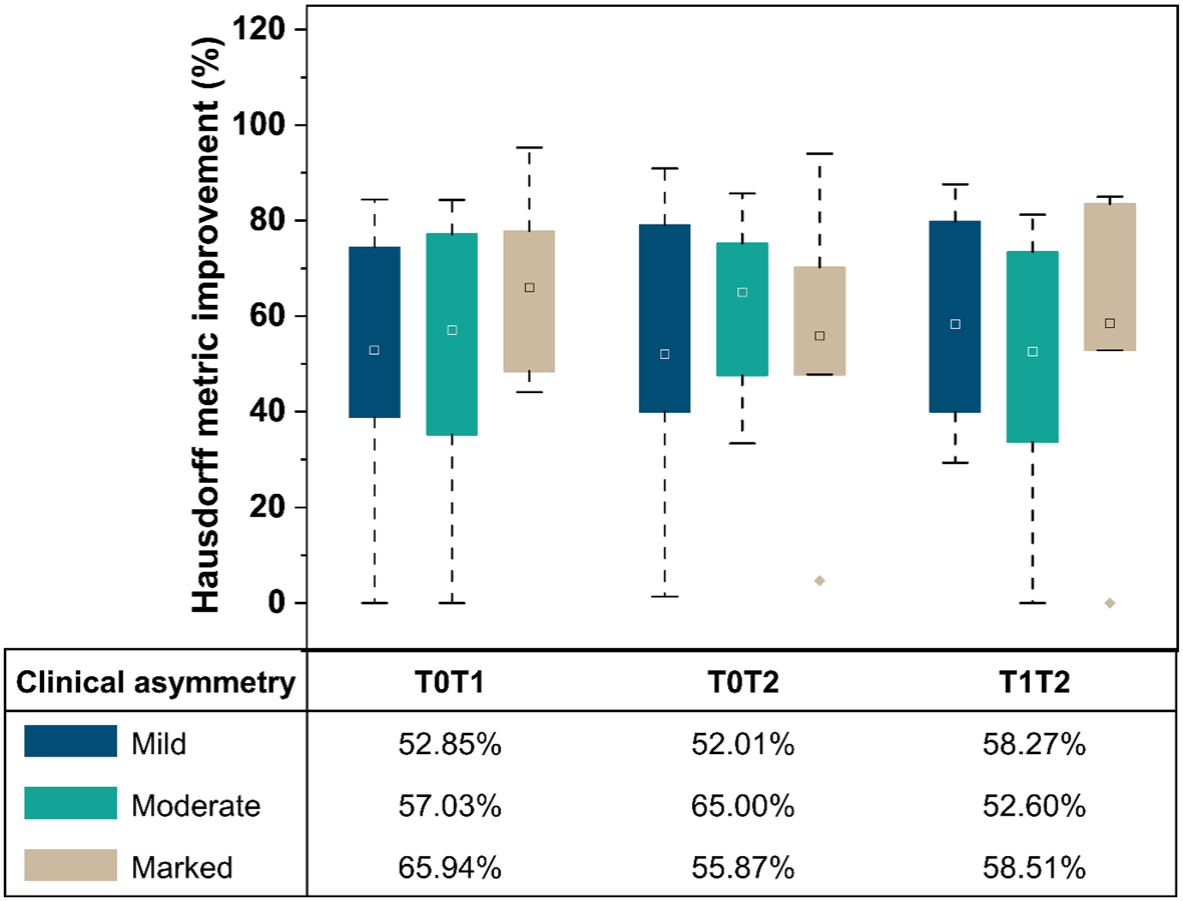
Goodness of alignment between pairs of serially recorded CBCT images expressed as percentage improvement with the orientation assignment using the Hausdorff metric. Clinical asymmetry groups clustered as mild, moderate, and marked. The x-axis tabulation shows the mean of the percentage improvement in the Hausdorff metric with and without the application of the proposed algorithm.

The k-medoid clustering was obtained with a silhouette coefficient of 0.746, indicating an acceptable cluster coherency based on clinically diagnosed asymmetry. The improvement in HD was similar between the three clusters showing no significant influence of the clinical asymmetry on the orientation assignment. The mean percentage reduction in each cluster shows that the goodness of alignment after orientation assignment improves by a minimum of 50% (Fig. 7, Table S2).

### Qualitative improvement in goodness of alignment

**Figure 8:**
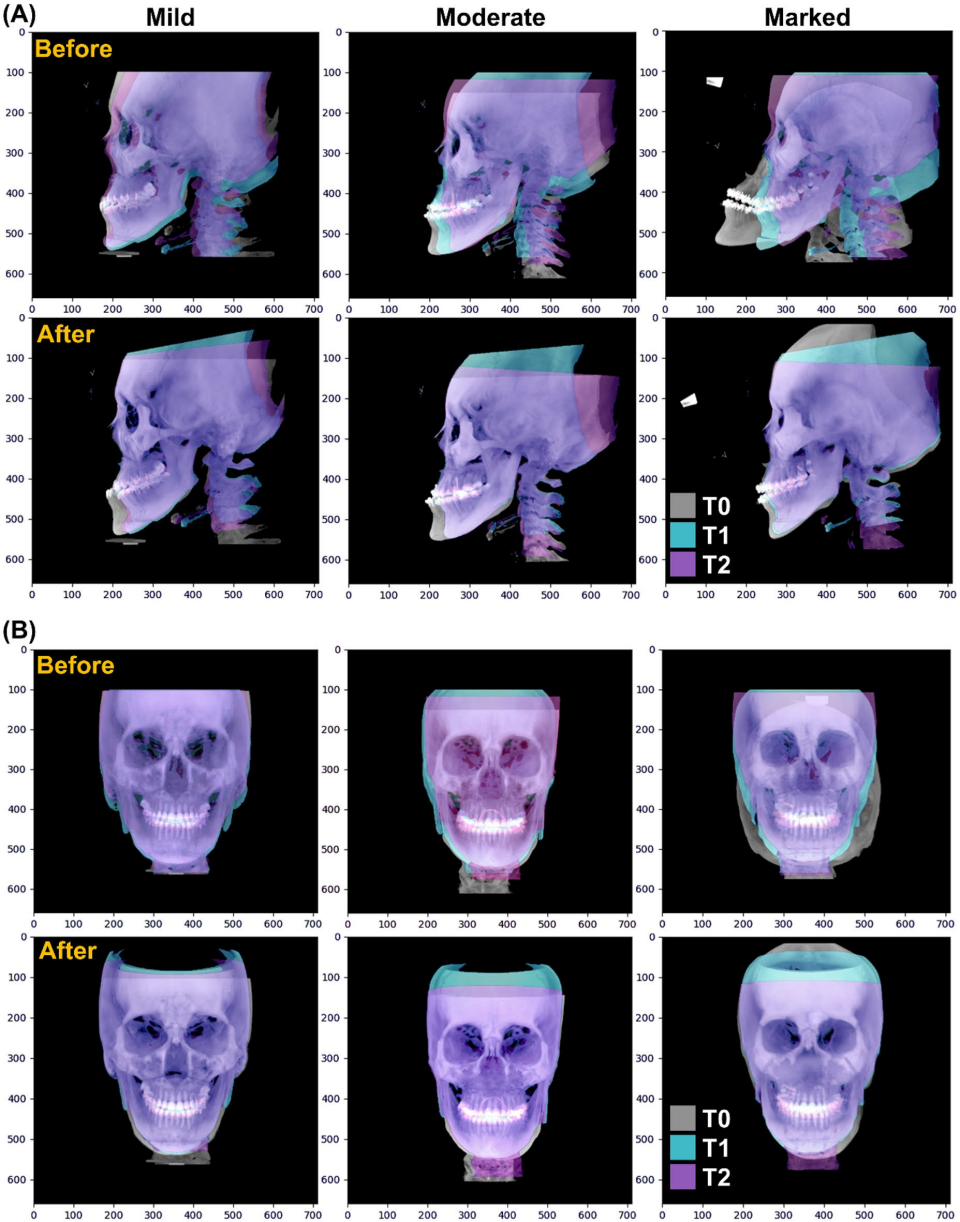
Qualitative visualization of the serial cone beam computed tomography volume. The columns represent three clusters of clinical asymmetries, i.e., mild, moderate, and marked. The (**A**) profile and (**B**) frontal images from three-time points (T0, T1, and T2) are presented as an overlay. The overlay images show an improvement in goodness after the proposed principal coordinate-based alignment is performed.

The qualitative visualization of the sequentially summed images before and after the assignment is shown in Fig. 8. A discernible and marked improvement can be observed in the cranial region for both frontal and profile views. The goodness of alignment can be seen in the forehead surface in Fig. 8A, extending from the identified nose tip to the superior surface. In Fig. 8B, the orbital region with well-demarcated internal orbital structures can be observed for all clusters of clinical asymmetries showing an improved agreement between serial CBCT volumes. The changes are most appreciable in the marked asymmetry group, where the T0 image presents a strikingly different orientation with poor reproduction of the posterior part of the skull. The group showed considerable improvement after the proposed protocol defined the new global axis.

## Discussion

Reproducible comparison of the longitudinal 3D CBCT images can enhance the overall clinical and research workflows. An evident challenge in analyzing serial 3D images from CBCT arises from the position of the head during capture and the variability in subsequent reorientation methods of imaging software^25^. Our study addressed the above issues by suggesting a landmark-free method that uses PCA for orientation assignments.

Previous studies have also proposed different algorithms for the alignment of images based on an idealistic dry skull model, heavy exposure, and high-resolution multi-slice computed tomography and external reference markers^4,9,26,27^. However, by using CBCT within the limits of a realistic clinical diagnostic environment, we could validate the direct use of the proposed method. The CBCT images present a high degree of variability between captures due to myriad factors, including age, sex, body morphology (BMI), breathing state, and position of the body during capture (e.g., Supine or standing)^7,28,29^. In addition, skeletal malocclusion affects the head position, with changes observed before and after treatment^14^. Another challenge with CBCT images is the voxel resolution and FOV. Cranial vault and base have been anatomically stable regions and have been preferentially suggested for standardizing the alignment of 3D images^27,30^. However, CBCT images acquired in dental practice do not consistently capture the skull’s vault, posterior and basal region. Hence, external fiduciary markers have been proposed^8^. The serial CBCT volumes presented in our study represent the three consecutive CBCT records of patients surgically treated for skeletal malocclusion for different magnitudes of asymmetry. This dataset helps validate our approach’s applicability in CBCT datasets corresponding to dental hospital settings. Moreover, as no additional marker placement is necessary, the computational approach can also be retrospectively applied to pre-existing records.

The use of PCA as a powerful technique in image processing has been commonly applied in a broad range of fields for the analysis of symmetry and alignment of shapes^31,32^. PCA prerequisites a well-defined object for which an eigenvector can be assigned. All regions of an image beyond the object are regarded as noise and interfere with the calculation accuracies^32^. The first step in our protocol factors both denoising and defining the ROI. The binarization with the Otsu method and 3D convex hull isolated the ROI from the medial aspect of the naso-orbital region. The stability of the upper third of the cranial region during growth and orthodontic therapy makes the ROI from the naso-orbital region a reproducible feature. Moreover, identifying the nose tip in the preprocessing step improves the definition of the principal axis for subsequent steps.

According to the results obtained, the protocol: (1) outlines a consistent method for defining an individualized global axis, (2) does not require a gold standard reference, and (3) is independent of user-defined configurations (e.g., craniometric landmark identification). The intermediary step, where the arrangement is performed within the computed ROI, improves accuracy. Studies have also proposed iterative closest point matching and mirroring methods using PCA as intermediate registration steps^30^. However, such methods rely on a registration reference and are more applicable in quantifying the symmetry of the 3D images. Notwithstanding the applicability of the registration algorithms, our protocol performs a comprehensive reorientation in 3D space. It can be considered as a step precursor to registration. With our protocol, the serial CBCT volumes show an improvement in HD metric by over 50%, which can be further enhanced by appending the registration methods. Hence, the orientation in 3D space using the method can be involved as a common step precursor to registration in the standard workflow.

The axis displacement in the CBCT images between individuals does not correspond with all axes. The resultant global axis may not vary significantly, and error within a few millimeters had been considered acceptable^14^. However, the axis displacement can be magnified when considering the differences in CBCT images from different time points for the same individual. It may be particularly relevant in cases where large displacement for surgical corrections is required. Our CBCT datasets, sub-divided based on the magnitude of clinical asymmetry, enabled us to evaluate the applicability of the proposed protocol. A notable improvement in the goodness of alignment could be observed across all clusters of clinical asymmetries.

The proposed method has potential for multiple clinical applications: (1) adaptation with the CBCT rendering software allowing an early-stage harmonization in longitudinal images; (2) determination of the orientation of the dental occlusion with craniofacial morphology (similar to facebow orientation); (3) precise and automated diagnosis of craniofacial asymmetries and (4) it may also help surgical treatment planning and follow up.

Despite the promising results of this work, the present approach has some limitations which might limit generalization and require interpretation with caution. The findings are based on a relatively limited data set from a single center. Although we believe the method would perform at par with different sources, it remains to be confirmed if the differences between the CBCT scanning equipment can influence the outcome. Secondly, the protocol was validated on an internally standardized hospital-based dose level and scan protocol. The dose-dependent variations can affect the resolution of the image and potentially induce sensitivity in the preprocessing step (Fig. 2).

Nonetheless, we believe that a more exhaustive and heterogenous sample with multiple timeframes from different CBCT sources and of patients with a larger age spectrum will aid in the generalization of our proposal.

## Conclusion

The findings from the present study elucidate the applicability of the computational method to address the fundamental challenge of harmonious skull orientation in longitudinally acquired CBCT images. The proposed protocol for 3D orientation produces an alignment of the skull rendered from CBCT images independently of user-defined landmarks. The computation was processed in two sequential steps, harmonizing the new global axis between serially captured CBCT images that could be validated with HD metric. Our study also validated the applicability of reorientation methodology in clinical cases with different ranges of skeletal asymmetry. Integration of the proposed algorithm as the preliminary step in CBCT evaluation will help improve the diagnostic and treatment plan workflow.

The proposed approach offers a promising improvement in addressing the fundamental step in the clinical and research-based use of CBCT data. By providing a harmonized starting point in 3D image visualization and analysis, our approach can improve the consistency of the existing methods (e.g., orientation with horizontal and vertical planes^33^). Additionally, for practical utilization, custom improvements can be built upon the proposed protocol toward achieving a standard harmonious workflow in the domain of craniomaxillofacial imaging.

## Supplementary Information

Supplementary Information.

## Data Availability

The data that support the findings of this study are available from the corresponding author upon reasonable request.
