# Supplementary material for: Reorientation methodology for reproducible head posture in serial cone beam computed tomography images
Source: Sci Rep. 2023 Feb 24;13:3220. doi: 10.1038/s41598-023-30430-4 (PMC9958024; doi:10.1038/s41598-023-30430-4)
Supplement: Supplementary file 1 — Supplementary Information. [file 41598_2023_30430_MOESM1_ESM.docx]

**Supplementary Information**

Reorientation Methodology for Reproducible Head Posture in Serial Cone Beam Computed Tomography Images.

**Utkarsh Mangal ^a, †^, Sung Min Lee ^b, †^, Seeyoon Lee ^a^, Jung-Yul Cha ^a^, Kee-Joon Lee ^a^, Hyung-Seog Yu ^a^, Hong Jung ^b^ and Sung-Hwan Choi ^a*^**

^a^Department of Orthodontics, Institute of Craniofacial Deformity, Yonsei University College of Dentistry, Seoul 03722, Korea; utkmangal@yuhs.ac (U. Mangal), seeyoonlee@yuhs.ac (S. Lee), jungcha@yuhs.ac.kr (J.Y. Cha), orthojn@yuhs.ac (K.J. Lee), yumichael@yuhs.ac (H.S. Yu) and selfexam@yuhs.ac (S.H. Choi)

^b^HDXWILL, Seoul 03162, Korea; kadama@naver.com (S.M. Lee), jh21star@hdx.co.kr (H. Jung)

Corresponding author

-Sung-Hwan Choi, Department of Orthodontics, Institute of Craniofacial Deformity, Yonsei University College of Dentistry, Seoul 03722, Korea

Email: selfexam@yuhs.ac (S.H.C)

† These authors contributed equally to this article

Table S1. The axes of rotation for the individual cone beam computed tomography records (n=29)

|  | T0 | T1 | T2 |
| --- | --- | --- | --- |
| 1 | [-0.3458, 0.0, -0.9383] | [-0.9802, 0.0, -0.1982] | [0.1142, 0.0, -0.9935] |
| 2 | [-0.9936, 0.0, -0.1133] | [-0.998, 0.0, 0.0632] | [-0.9957, 0.0, 0.0927] |
| 3 | [0.5189, 0.0, -0.8548] | [-0.8638, 0.0, -0.5039] | [-0.7254, 0.0, -0.6884] |
| 4 | [-1.0, 0.0, -0.0083] | [-0.5368, 0.0, -0.8437] | [-0.9994, 0.0, -0.0359] |
| 5 | [-0.9337, 0.0, -0.3581] | [-0.5223, 0.0, 0.8528] | [-0.9988, 0.0, 0.0486] |
| 6 | [-0.8713, 0.0, -0.4908] | [-0.8276, 0.0, -0.5613] | [-0.8252, 0.0, -0.5649] |
| 7 | [-0.999, 0.0, 0.045] | [-0.6469, 0.0, 0.7626] | [-0.9337, 0.0, -0.358] |
| 8 | [-0.9823, 0.0, -0.1873] | [-0.8683, 0.0, -0.496] | [-0.9527, 0.0, -0.3039] |
| 9 | [-0.8986, 0.0, -0.4388] | [-0.8939, 0.0, -0.4482] | [-0.8949, 0.0, -0.4462] |
| 10 | [0.9437, 0.0, -0.3308] | [0.9627, 0.0, -0.2706] | [0.7711, 0.0, -0.6368] |
| 11 | [-0.9996, 0.0, 0.0273] | [-0.9781, 0.0, 0.2079] | [-0.9585, 0.0, -0.2851] |
| 12 | [-0.9931, 0.0, 0.1172] | [-0.9972, 0.0, -0.0747] | [-0.8926, 0.0, -0.4509] |
| 13 | [-0.4657, 0.0, -0.885] | [-0.9848, 0.0, -0.1734] | [-0.9532, 0.0, -0.3024] |
| 14 | [-0.2451, 0.0, -0.9695] | [-0.9999, 0.0, 0.0141] | [-0.9999, 0.0, -0.0116] |
| 15 | [-0.9999, 0.0, -0.0164] | [-0.9406, 0.0, -0.3396] | [0.4742, 0.0, -0.8804] |
| 16 | [0.9845, 0.0, -0.1754] | [0.4986, 0.0, -0.8668] | [-0.6895, 0.0, -0.7243] |
| 17 | [-0.9955, 0.0, 0.0945] | [-0.9945, 0.0, -0.1047] | [-0.9876, 0.0, 0.1572] |
| 18 | [0.7452, 0.0, -0.6668] | [-0.7778, 0.0, -0.6285] | [-0.9737, 0.0, -0.228] |
| 19 | [0.9001, 0.0, -0.4357] | [0.988, 0.0, -0.1544] | [0.8617, 0.0, -0.5075] |
| 20 | [-0.9959, 0.0, -0.0899] | [-1.0, 0.0, -0.0016] | [-0.9951, 0.0, 0.0987] |
| 21 | [-0.8653, 0.0, -0.5013] | [-0.9446, 0.0, -0.3281] | [-0.8965, 0.0, -0.4431] |
| 22 | [-0.6645, 0.0, -0.7473] | [-0.9964, 0.0, -0.0842] | [-0.9468, 0.0, -0.3219] |
| 23 | [0.5259, 0.0, -0.8505] | [-0.5724, 0.0, -0.82] | [-0.5036, 0.0, -0.8639] |
| 24 | [-0.9667, 0.0, -0.2558] | [-0.9713, 0.0, -0.2377] | [-0.991, 0.0, -0.134] |
| 25 | [1.0, -0.0, 0.0034] | [0.858, 0.0, -0.5136] | [0.9874, -0.0, 0.158] |
| 26 | [-0.9567, 0.0, -0.291] | [-0.9387, 0.0, -0.3448] | [-0.6788, 0.0, -0.7343] |
| 27 | [-0.7116, 0.0, 0.7026] | [-0.739, 0.0, 0.6737] | [-0.8961, 0.0, 0.4439] |
| 28 | [-0.4146, 0.0, -0.91] | [-0.8584, 0.0, -0.513] | [0.9564, 0.0, -0.2921] |
| 29 | [-0.9647, 0.0, -0.2634] | [-0.9975, 0.0, 0.0713] | [-0.9184, 0.0, 0.3957] |

Table S2. Matching improvement in the Hausdorff metric before and after the reorientation protocol.

| **Cluster^1^** | | **Before** | | | **After** | | |
| --- | --- | --- | --- | --- | --- | --- | --- |
|  |  | **T0-T1** | **T0-T2** | **T1-T2** | **T0-T1** | **T0-T2** | **T1-T2** |
| 1 | 7.3274 | | 3.7204 | 4.3077 | 1.17 | 0.78 | 0.6755 |
| 1 | 7.5018 | | 4.29 | 3.5316 | 1.17 | 0.39 | 0.8721 |
| 1 | 3.1443 | | 2.3723 | 4.3253 | 0.9553 | 2.34 | 2.3723 |
| 1 | 5.9146 | | 8.7207 | 5.085 | 3.6167 | 4.7446 | 3.5531 |
| 1 | 1.2935 | | 1.17 | 1.17 | 0.9553 | 0.78 | 0.5515 |
| 1 | 1.7 | | 4.68 | 6.2886 | 0.78 | 0.6755 | 0.78 |
| 1 | 4.3077 | | 2.34 | 2.73 | 1.1031 | 1.17 | 0.5515 |
| 1 | 1.56 | | 2.1361 | 1.56 | 0.8721 | 1.2333 | 1.1031 |
| 1 | 0.78 | | 1.95 | 1.95 | 0.78 | 1.17 | 1.17 |
| 2 | 4.9485 | | 6.3487 | 1.9106 | 1.17 | 1.1031 | 0.6755 |
| 2 | 4.3077 | | 3.5316 | 1.56 | 0.6755 | 1.17 | 1.17 |
| 2 | 4.0904 | | 2.73 | 6.7437 | 1.608 | 0.39 | 1.6546 |
| 2 | 1.2333 | | 3.12 | 4.0904 | 0.8721 | 0.78 | 0.8721 |
| 2 | 0.78 | | 1.17 | 1.2333 | 0.5515 | 0.78 | 0.39 |
| 2 | 4.29 | | 2.2404 | 2.73 | 1.56 | 1.17 | 0.78 |
| 2 | 2.1002 | | 3.5956 | 2.587 | 2.2404 | 3.51 | 2.7577 |
| 2 | 1.2333 | | 1.56 | 0.5515 | 2.73 | 1.2935 | 2.9702 |
| 2 | 2.7852 | | 4.7285 | 2.0265 | 0.78 | 1.17 | 1.17 |
| 2 | 1.95 | | 1.56 | 0.78 | 0.5515 | 0.39 | 0.78 |
| 2 | 7.0416 | | 7.41 | 0.5515 | 4.1458 | 4.0153 | 0.5515 |
| 2 | 3.5531 | | 2.1002 | 2.4666 | 0.78 | 1.1031 | 0.9553 |
| 2 | 0.78 | | 3.51 | 3.1443 | 0.78 | 0.8721 | 1.17 |
| 2 | 2.4972 | | 3.9 | 6.24 | 0.5515 | 1.17 | 1.17 |
| 3 | 3.51 | | 1.2935 | 4.7124 | 0.78 | 0.6755 | 0.78 |
| 3 | 3.12 | | 1.2935 | 2.1002 | 1.608 | 1.2333 | 0.78 |
| 3 | 3.9 | | 3.51 | 3.5316 | 1.17 | 1.1031 | 1.17 |
| 3 | 1.95 | | 1.56 | 1.17 | 0.78 | 0.78 | 1.169 |
| 3 | 20.28 | | 27.624 | 7.8 | 0.9553 | 1.6546 | 1.17 |
| 3 | 1.56 | | 2.6162 | 1.17 | 0.8721 | 0.78 | 0.5515 |

^1^k-medoid clustering (silhouette coefficient=0.746)
